# Supplementary figures and images for: Comparison of pediatric scoring systems for mortality in septic patients and the impact of missing information on their predictive power: a retrospective analysis
Source: PeerJ. 2020 Oct 5;8:e9993. doi: 10.7717/peerj.9993 (PMC7543722; doi:10.7717/peerj.9993)

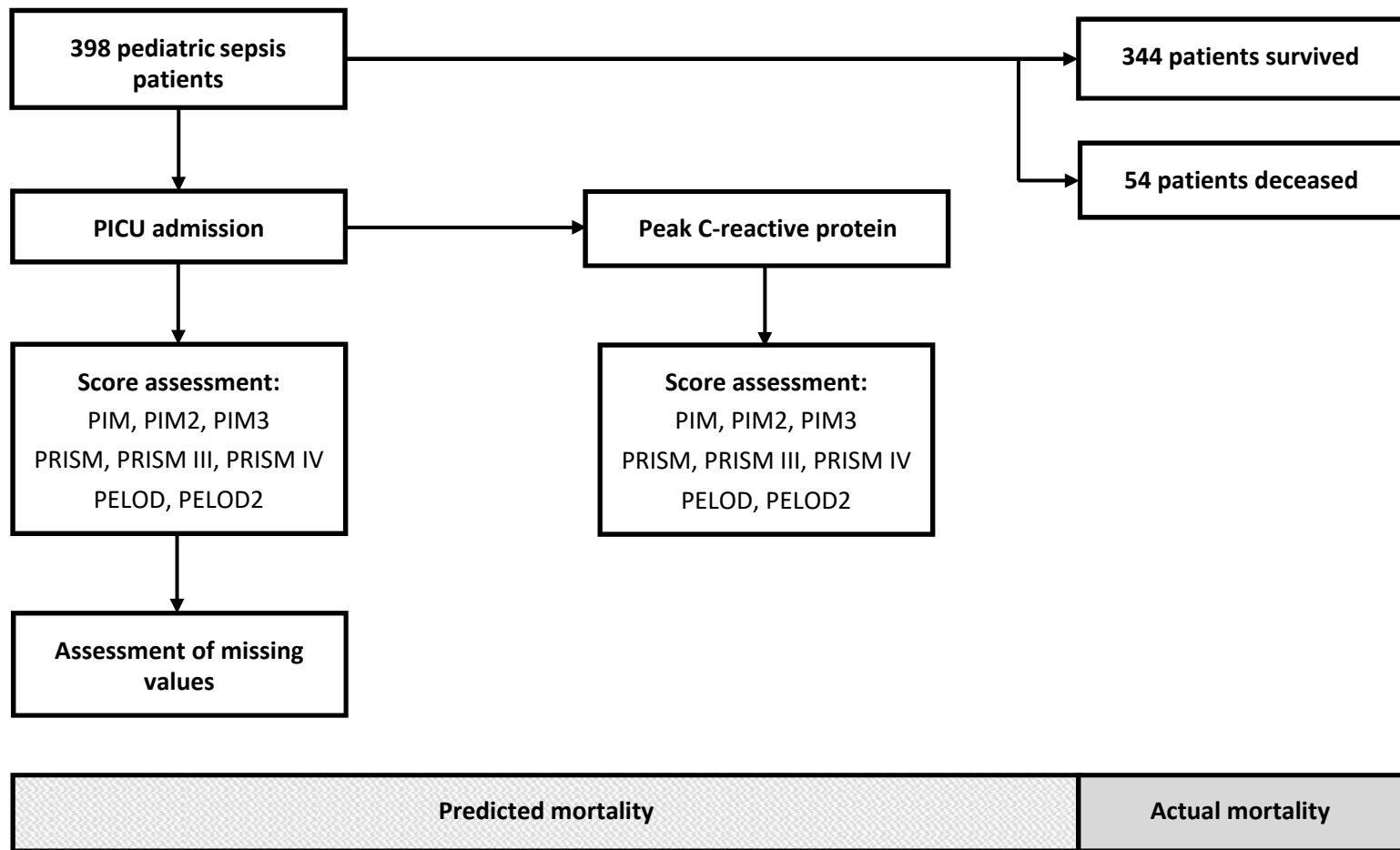

Supplement: Supplemental Information 1 — PRISM, PRISM III, PRISMIV, PIM, PIM2, and PIM3 as well as PELOD and PELOD2 scores were assessed at PICU admission and peak C-reactive protein. Predicted mortality was then compared with actual mortality. [file peerj-08-9993-s001.pdf]
